# mRNA Vaccines: Why Is the Biology of Retroposition Ignored?

**DOI:** 10.3390/genes13050719

**Published:** 2022-04-20

**Authors:** Tomislav Domazet-Lošo

**Affiliations:** 1 Laboratory of Evolutionary Genetics, Division of Molecular Biology, Ruđer Bošković Institute, Bijenička Cesta 54, HR-10000 Zagreb, Croatia; tdomazet@irb.hr; 2 School of Medicine, Catholic University of Croatia, Ilica 242, HR-10000 Zagreb, Croatia

**Keywords:** mRNA vaccines, retroposition, L1 elements, LINE-1, retrotransposons, retrocopy, parental genes, genome integration, insertional mutagenesis

## Abstract

The major advantage of mRNA vaccines over more conventional approaches is their potential for rapid development and large-scale deployment in pandemic situations. In the current COVID-19 crisis, two mRNA COVID-19 vaccines have been conditionally approved and broadly applied, while others are still in clinical trials. However, there is no previous experience with the use of mRNA vaccines on a large scale in the general population. This warrants a careful evaluation of mRNA vaccine safety properties by considering all available knowledge about mRNA molecular biology and evolution. Here, I discuss the pervasive claim that mRNA-based vaccines cannot alter genomes. Surprisingly, this notion is widely stated in the mRNA vaccine literature but never supported by referencing any primary scientific papers that would specifically address this question. This discrepancy becomes even more puzzling if one considers previous work on the molecular and evolutionary aspects of retroposition in murine and human populations that clearly documents the frequent integration of mRNA molecules into genomes, including clinical contexts. By performing basic comparisons, I show that the sequence features of mRNA vaccines meet all known requirements for retroposition using L1 elements—the most abundant autonomously active retrotransposons in the human genome. In fact, many factors associated with mRNA vaccines increase the possibility of their L1-mediated retroposition. I conclude that is unfounded to a priori assume that mRNA-based therapeutics do not impact genomes and that the route to genome integration of vaccine mRNAs via endogenous L1 retroelements is easily conceivable. This implies that we urgently need experimental studies that would rigorously test for the potential retroposition of vaccine mRNAs. At present, the insertional mutagenesis safety of mRNA-based vaccines should be considered unresolved.

## 1. Introduction

The research and development of mRNA-based therapeutics gained momentum with the onset of the COVID-19 pandemics. Currently, two mRNA vaccines against SARS-CoV-2 (BioNTech/Pfizer BNT162b2 and Moderna mRNA-1273) are approved for use in the general population in many countries, e.g., [1,2], and several others are under development [3,4,5]. It was often suggested that the main advantage of mRNA-based vaccines, compared to the more conventional approaches is the possibility of their rapid development and large-scale deployment [6,7], which are both desirable properties in pandemic situations. The statement that vaccine mRNAs do not pose a risk for genome integration, e.g., [6,8,9,10,11,12], and consequently, that there is no insertional mutagenesis risk, is another commonly listed advantage of mRNA-based vaccines, especially when contrasted with the safety profile of DNA-based therapeutics [10,12,13]. This claim prompted me to look more carefully into the mRNA vaccine literature to find a rationale for it. Surprisingly, I was not able to track down any experimental or theoretical study that specifically addresses the possibility of genome integration of mRNA therapeutics.

This shortage of relevant studies is reflected in numerous reviews [4,5,6,9,10,14,15,16,17,18], book chapters on the mRNA vaccines [13,19,20,21,22] and documents of international organizations [23,24,25], which often state that mRNA vaccines do not pose the risk for genome integration but do not cite any references in support of this idea. Occasionally, some citations are embedded, e.g., [15,22,26,27], but unfortunately, they are circular as they point to similar unsupported statements [6,10,21,28,29,30]. This signals that the idea of vaccine mRNA’s resistance to genome integration behaves like a meme that self-replicates in the literature and, therefore, it should not be considered reliable scientific information. Undoubtedly, there is always a possibility that my literature search missed some important work; however, other researchers also notice, although without going into details, the shortage of studies that explicitly deal with the possibility of vaccine mRNA genome integration [13,31,32,33,34]. In the absence of such studies, statements such as “In addition, IVT mRNA-based therapeutics, unlike plasmid DNA and viral vectors, do not integrate into the genome and therefore do not pose the risk of insertional mutagenesis.” [10], “First, safety: as mRNA is a non-infectious, non-integrating platform, there is no potential risk of infection or insertional mutagenesis.” [6] or “mRNA-based vaccines avoid the risk of integration with the host cell genome …” [24,25] obviously go against sound scientific epistemology.

Besides the lack of references, the argumentation line for the claim that the genome integration of vaccine mRNA molecules is not possible, or is negligible, is rather limited in the vast majority of papers. Many of them simply state that vaccine mRNA cannot integrate into the host genome without explaining why this is not possible [3,10,12,19,20,21,22,26,30]. Others shortly describe that vaccine mRNAs remain in the cytoplasm of the host cells—in contrast to DNA-based vaccines, which must enter the nucleus to be effective—and thus do not have the opportunity to change the genome [4,9,18,27,35].

Recently, some papers argued that the relatively short persistence of mRNA makes genome integration of mRNA vaccines improbable [4,13,27]. However, some of them also recognize the possibility of genome integration if vaccine mRNA is reverse-transcribed in the host cells [4,13,31]. Although human endogenous retroviruses (HERVs) and retroviral infections (e.g., HIV) are mentioned as a possible source of enzymes for reverse transcription and genome integration, the common conclusion is that the integration risk is still highly unlikely [4,31]. In contrast, some authors are more cautious and suggest that investigation may be needed to clarify whether vaccine mRNA integration can occur [13].

## 2. The Biology of Retroposition

**Figure 1:**
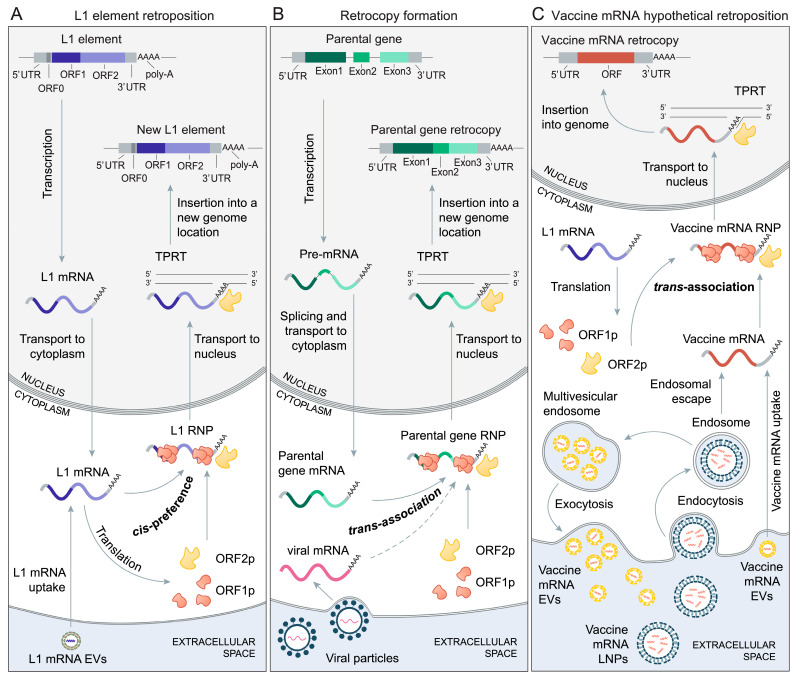
L1-mediated retroposition. (**A**) Retroposition cycle of L1 elements. An active L1 element is transcribed in the nucleus and the resulting L1 mRNA is transported to the cytoplasm where it undergoes translation [44,45]. L1 mRNA codes for ORF1 and ORF2 proteins, which preferentially associate with L1 mRNA (*cis*-preference) to form L1 ribonucleoprotein particle (L1 RNP) [44,45,46]. ORF1p is an RNA binding protein with chaperone activity, while ORF2p functions as reverse transcriptase and endonuclease [47,48]. By a yet unresolved mechanism, L1 RNP, which contains at least L1 mRNA and ORF2p, enters the nucleus. In the nucleus, L1 mRNA is reverse-transcribed and integrated into the genome via the process of target-primed reverse transcription (TPRT) [45,47,48,49]. The retroposition mechanism relies on the binding of ORF2p to the L1 mRNA poly-A tail [48,50,51,52]. There is some evidence that the cells could uptake extracellular vesicles (EVs) containing L1 mRNA, which can then undergo translation and retroposition [53]. (**B**) L1-mediated retroposition of endogenous coding genes and L1-mediated retroposition of viral mRNAs. A parental protein-coding gene is transcribed in the nucleus. The resulting pre-mRNA is processed and mature parental gene mRNA is then transported to the cytoplasm. L1 proteins (ORF1p and ORF2p) interact with parental gene mRNA via the process termed *trans*-association to form a parental gene ribonucleoprotein particle (parental gene RNP) [36,45,46,49]. Similar to L1 RNP, a parental gene RNP enters the nucleus where the parental gene mRNA, through TPRT, is reverse-transcribed and integrated into the genome. The poly-A tail of parental gene mRNA plays a crucial role in this process [36,50,51,52]. By a similar process, mRNA molecules that stem from non-retroviral RNA viruses could be integrated into the genome. Examples include the integration of mRNAs from bornaviruses [42,43] and probably coronaviruses [54,55]. (**C**) Hypothetical L1-mediated retroposition of vaccine mRNA. Vaccine mRNA formulated in lipid nanoparticles (LNPs) enter the cell via endocytosis [1,2,6,10,56]. A fraction of the vaccine mRNA enters the cytosol via endosomal escape, while the rest of the vaccine mRNA undergoes degradation in endosomes [56] or is repackaged in multivesicular endosomes into extracellular vesicles (EVs) and secreted back into the extracellular space [57]. The neighboring or distant cells can uptake vaccine mRNA from these EVs [57,58]. L1 proteins (ORF1p and ORF2p) interact with vaccine mRNA via a process termed *trans*-association to form a vaccine mRNA ribonucleoprotein particle (vaccine mRNA RNP) [36,45,46,49]. Like L1 and parental gene RNPs, a vaccine mRNA RNP enters the nucleus where the vaccine mRNA, through TPRT, is reverse-transcribed and integrated into the genome. The poly-A tail of vaccine mRNA plays a crucial role in this process [36,50,51,52].

Nevertheless, this discussion within the vaccinology field on the vaccine mRNA genome integration risks is rather brief and surprisingly incomplete, as it does not consider the accumulated knowledge on the biology of retroposition [36,37,38,39,40]. In many eukaryotes, the cellular mRNAs of various genes are endogenously reverse-transcribed and reintegrated into the genome, yielding their retrocopies (Figure 1B) [36,38,39,40]. This process of mRNA-mediated gene duplication is highly frequent in therian mammals [41] and is best studied in primates and mice [36,37,38,40]. Besides endogenous genes, the retroposition of mRNAs coming from non-retroviral RNA viruses (bornaviruses) is also reported [42,43]. Of note, the term retrocopy is often interchanged with other related terms, such as processed pseudogenes, retrotransposed pseudogenes, retropseudogenes, retroposed gene copies, retroCNVs and retrogenes, as the terminology related to retroposition is not yet fully settled [38,39].

Depending on the annotation methodology, the estimated number of retrocopies in the human genome varies, but the figures in most studies are approximately 8000 [38,39,59,60]; these retrocopies are derived from around 2500 parental genes [59,61], i.e., genes whose mRNAs are reverse-transcribed and integrated into the genome (Figure 1A,B). These values are similarly high in all screened therian mammals and reflect endogenous retroposition activity during the ~200 My of their evolution [41,61]. However, the continuous activity of retroposition is also apparent in extant human populations, where substantial polymorphism of novel retrocopies is revealed [37,60,62,63,64]. For instance, it was estimated that an individual harbors on average six novel retrocopies that are absent from the human reference genome, and that these retrocopies were derived from the pool of 503 unique parental genes [37]. These values indicate a rather high retroposition activity in present human populations.

A recent study in mice suggested that the actual rate of retrocopy generation in extant populations is even higher and possibly similar between humans and mice [40], and hence, it is not surprising that retrocopy variation is detected in medical contexts [65,66]. However, it is also suggested that due to the use of unoptimized analytical pipelines, many retrocopies have often been overlooked in routine genetic testing [40,65]. At present, there are several documented cases of retrocopy emergence related to diseases in animals [49,65,67], and one case of pathogenic retrocopy in humans [49,65,68,69], but more could potentially be discovered [40]. Actually, it seems that retrocopy variation in human populations might be more phenotypically relevant and population-specific than single nucleotide polymorphisms [37,40] and that most newly transposed retrocopies have a deleterious impact [40]. All of this suggests that the mutation load coming from the retroposition activity in extant human populations is medically relevant.

Regardless of the initial selective purge [40], retrocopies are a source of novel genes with adaptive significance that contribute to human biology and health [36,39]. Retrocopies have been viewed as the unfunctional remnants of evolutionary turnover, termed processed pseudogenes [39], mainly because it was presumed that retrocopies inherently lack transcription-driving elements and, thus, could not be transcribed [39,40,41]. A similar argument is recently raised in the vaccinology field when the possibility of vaccine mRNA genome integration and its impact on phenotypes is discussed [13]. However, after it was realized that most regions of a mammalian genome are transcribed [70,71,72] and that retrocopies could easily gain their own regulatory elements [36,38,40,41], it has become apparent that most retrocopies show evidence of transcription [38,40,41].

These transcribed retrocopies are thus the source of evolutionary innovations, as they could be further transformed into novel protein-coding or RNA retrogenes [36,38,41,73]. Approximately several hundred RNA and several hundred protein-coding retrogenes are estimated to be active in humans and mice [36,38]. For most of them, the functional significance has yet to be determined, but some are known to be human disease genes [74,75,76] or to have discernible phenotypes [36,38].

Many of the retrocopies I have discussed so far are vertically transmitted through the germline, but mRNA retroposition also occurs in somatic tissues. Somatic retroposition is substantially less studied, but it is known to be common in cancer tissues [62,77,78,79,80] and to occur during early development [68,69]. However, the activity of endogenous retroelements that drive retroduplication in humans suggests that mRNA retroposition events should be found in other somatic tissues as well (see below). This indicates that retrocopies continuously reshape the human genome, not only at the population level and deeper evolutionary time scale but also in somatic tissues during individual development. It is therefore important to consider the endogenous drivers of retroposition in humans when the probability of vaccine mRNA genomic integration is evaluated.

## 3. The Mechanisms of Retrocopy Formation

The mechanism that leads to the formation of retrocopies in a human lineage is relatively well studied and predominantly includes long interspersed element-1 (LINE-1 or L1, Figure 1A) retrotransposons [36,38,40,46,81], albeit there is some evidence that retroposition through long terminal repeat (LTR) retrotransposons is also possible [38,81]. However, very surprising quotes such as “The only known mechanism by which RNA can integrate into the host genome is in the presence of a retrovirus particle containing reverse transcriptase.” [23] reveal that the vaccinology field, for an unclear reason, is unaware of the existence and significance of L1-driven retroposition in humans. To bridge this gap between the fields, and to set the stage for the discussion on the possibility of vaccine mRNA retroposition, I first provide here an overview of the L1-dependent retroposition mechanism.

L1 retroelements are around 6 kb long, make up 17 percent of the human genome and around one hundred of them are active in spreading their copies in the genome by means of retroposition of their own mRNA (Figure 1A) [44,45,49,82,83,84,85]. When transcribed, L1 produces bicistronic mRNA that codes for two proteins: ORF1p is an RNA binding protein with chaperone activity, while ORF2p functions as reverse transcriptase and endonuclease [44,45,47,48,49,84,85]. Together with an L1 mRNA, these proteins assemble in the cytoplasm into an L1 ribonucleoprotein particle (L1 RNP), which can then enter the nucleus (Figure 1A) [44,45,47,48,49,84,85].

In the nucleus, L1 mRNA is eventually reverse-transcribed and integrated into the genome at A/T-rich consensus target sites via the process termed target-primed reverse transcription (TPRT) (Figure 1A) [45,47,48,49]. In the antisense direction, L1 also codes for ORF0p, which is a small peptide that localizes in the nucleus and enhances the efficiency of retrotransposition [49,86]. During the L1 lifecycle, diverse host proteins interact with L1 RNPs via promoting or suppressing their retrotransposition [49,87]. The L1 protein machinery preferentially targets their encoding mRNA (*cis*-preference), but it can also mobilize a variety of other RNAs present in the cell (*trans*-association), including non-autonomous mobile elements (Alu, SVA), splicesomal RNAs and diverse protein-coding mRNAs (Figure 1B) [45,46,49,83,88].

This relaxed retroposition behavior of L1 elements, which allows for the mobilization of various mRNAs through *trans*-association, is responsible for the massive accumulation of non-autonomous mobile elements and retrocopies in genomes (Figure 1B). The question arises regarding how L1 elements achieve such promiscuous performance. The underlying reason for such behavior is linked to the L1 retroposition mechanism that is contingent on ORF2p binding to the poly-A tail during RNP formation in the cytoplasm (Figure 1) [50,51]. Subsequently in the nucleus, genome integration also relies on the poly-A tail, which permits flexibility in DNA priming at the target site during the TPRT process [48,52]. Given that poly-A tails are unspecific low-complexity sequences that are almost ubiquitously present at the 3′ ends of cellular mRNAs [89], this implies that, in principle, every mRNA could be a target of L1 protein machinery and undergo the TPRT process (Figure 1C).

However, the complete lack of retroposition specificity would significantly lower the fitness of L1 elements and compromise their parasitic proliferation in the genomes. To avoid this scenario, L1 elements managed to preferentially target their own mRNA, regardless of the poly-A tail dependence [46,90,91]. A popular model that tries to explain the mechanisms of this *cis*-preference envisages that, during translation, emerging L1 proteins associate immediately with their encoding mRNA at the ribosome [44,47,50,92]. Obviously, this or a similar process ensures the balance between the parasitic reproduction of L1 elements and the occasional mobilization of diverse mRNAs by *trans*-association via poly-A tracts (Figure 1).

## 4. L1 Elements in Germline and Soma

The possibility of vaccine mRNA integration and its resulting phenotypic consequences highly depend on the overall dynamics of L1 retroelements in the human body, which is a topic that I consider in this section.

L1 activity is an important contributor to genetic variation within and between individuals with implications for evolution and disease in humans [45,85,93]. Interaction between the host genome and L1 elements is multilayered with beneficial and detrimental effects on the host fitness [93,94,95,96,97,98]. For this reason, the host cells evolved various mechanisms to keep their activity in balance [93,96,99,100,101,102,103,104]. Regardless of these host protection mechanisms, a new retroposition event mediated by L1 elements must occur in the germline to be passed to the next generation [97].

The mere presence of numerous vertically inherited L1 elements, non-autonomous mobile elements and retrocopies in human genomes provides direct evidence that their mobilization repeatedly occurs in the germline [99]. It was also well established that L1 activity contributes to ongoing germline mutagenesis [105,106]. However, the precise dynamics of retroposition during the germline lifecycle are less clear [96,97,107,108]. The current data suggest that L1 elements show expression and retroposition activity in testes [96,105,106,109], spermatozoa [110,111], ovaries [105,106], oocytes [112] and early embryos [97,99,105,107,108,113].

Although it was initially thought that L1 elements are mainly active in the germline, accumulated evidence suggests that they also should be considered an endogenous mutagen in somatic tissues [99,100,106,114]. L1 elements are expressed in diverse human somatic tissues, including liver, spleen, adrenal glands, lungs, heart and brain [106]; lymphoblastoid cell lines [115]; platelets; megakaryocyte; and T cells [98]. Expression and retroposition activity of L1 elements was detected in vascular endothelial cells as well [109,116]. However, somatic L1 retroposition has been extensively studied only in the brain, cancer tissues and the gastrointestinal tract [45,78].

During both embryonic and adult neurogenesis, L1 retroposition activity generates significant neuronal mosaicism [60,99,117,118,119,120,121], which further increases in neurological disorders [121,122]. L1 retroposition occurs in diverse cell types of the central nervous system, including glial cells, neuronal progenitor cells, differentiating neurons and mature non-dividing neurons [118,121,123,124,125,126]. It is speculated that L1-driven somatic mosaicism may alter the functional properties of neural cells and that many of them may contain a unique genome [118,126]. However, the biological and medical significance of this mosaicism is not fully clear [120,121,122].

L1 elements are also highly expressed in many human cancers, where they function as an endogenous mutagen and can be responsible for driving mutations in tumorigenesis [84,85]. Epithelial cancers seem to be particularly prone to L1 retroposition [45,78]. Interestingly, L1 insertions are found in tumor cells, as well as normal cells of liver, stomach, colon and esophagus [127,128,129,130], suggesting widespread somatic activity of L1 elements in the gastrointestinal tract. In general, somatic L1 retroposition is highly ontogeny dependent and strongly increases with advanced age due to L1 transcriptional derepression [104,131]. In addition to endogenous regulation, the activity of L1 elements is sensitive to exogenous signals and could be induced by numerous environmental factors [93,99,100,114,122]. Taken together, it is clear that human germinative and many somatic cells have a lasting potential for L1-mediated retroposition via *cis*-preference and *trans*-association (Figure 1).

## 5. Vaccine mRNAs and Retroposition

Evidently, various mRNAs in humans could be reverse-transcribed and integrated into the genome via L1 retroelements with negative effects on fitness. However, this does not readily imply that this will occur to vaccine mRNAs. A definitive answer will come from experiments and population monitoring, but for now, it is helpful to consider their described properties and evaluate them against the L1 retroposition mechanism (Figure 1). The active substance of the BNT162b2 vaccine is a 4284-nucleotide-long synthetic mRNA molecule that contains N1-methylpseudouridine (m1Ψ), a modified nucleoside that substitutes naturally occurring uridine [1,132,133]. This nucleoside modification reduces the innate immune response to exogenous mRNA molecules and enhances their translation (Table 1) [6,134,135,136]. Structurally, BNT162b2 mRNA consists of a 5′ cap analog, a 5′ untranslated region, a codon-optimized SARS-CoV-2 spike-protein-coding sequence, a 3′ untranslated region and a 110-nucleotide poly-A tail [1,56,132,133]. These structural elements follow the usual eukaryotic mRNA architecture and help to increase RNA stability and translational efficiency of mRNA vaccines (Table 1) [6,10,28,133]. In contrast to BNT162b2, the exact mRNA sequence of the mRNA-1273 vaccine seems not to be publicly disclosed [56]. However, its general design is similar to BNT162b2 mRNA, including the use of m1Ψ instead of uridine, the presence of a 5′ cap structure, a 5′ untranslated region, a codon-optimized spike-protein-coding sequence, a 3′ untranslated region and a poly-A tail [2,137].

From the perspective of their sequence arrangement, BNT162b2 and mRNA-1273 mRNA synthetic molecules appear to be suitable targets for L1 retroposition in *trans* because they structurally and functionally mimic the architecture of native mRNAs that occur in the cytoplasm of eukaryotic cells (Table 1) [6,10]. In this regard, probably the most important sequence feature is their poly-A tail, which is known to be required for L1-mediated retroposition (Figure 1, Table 1) [51]. However, the available information on the vaccine mRNA engineering logic reveals that vaccine mRNAs were not specifically constructed to avoid capture by the L1 retroposition machinery [1,2,6,10,56]. In fact, it seems that no study in the mRNA vaccine field considered this possibility, e.g., [4,6,10,13,31]. For instance, the poly-A tail of BNT162b2 mRNA contains a 10-nucleotide-long linker sequence that is flanked by 30- and 70-nucleotide-long adenosine tracts [132]. Nevertheless, this poly-A tail modification, which helps in increasing translational efficiency [133,147], is unlikely to affect the retroposition propensity of the vaccine mRNA because only nucleotide changes directly neighboring the 3′ end of the poly-A tail are known to have a significant impact on the L1 retroposition mechanism [51,52,102]. Moreover, non-adenosine nucleotides at the 3′ end of the poly-A tail are generally avoided in mRNA therapeutics, as they hamper translational efficiency [148]. Similarly, because of the total number of modified nucleotides per mRNA molecule, the m1Ψ ribonucleoside modification is perhaps the most striking artificial feature of the vaccine mRNAs; however, these types of ribonucleoside modifications generally do not prevent reverse transcription [149].

## 6. Parental Genes and BNT162b2

In the comparative context, genes known to actively generate retrocopies (parental genes) in extant populations (Figure 1B) are the best reference to assess general mRNA sequence trends related to retroposition. However, the collective properties of parental genes have not been extensively analyzed. Some studies report that parental genes are enriched in translation, ribosome, intracellular lumen and cell-division-related functional categories [37,62,64] and that they have a weak tendency to be highly expressed [37], but a more detailed analysis is still missing. It is helpful then to explore here some basic sequence properties of mRNAs transcribed from parental genes that are known to actively generate retrocopies in extant populations [37,40] and then to relate this information to the vaccine mRNA sequence that is publicly available (i.e., BNT162b2).

**Figure 2:**
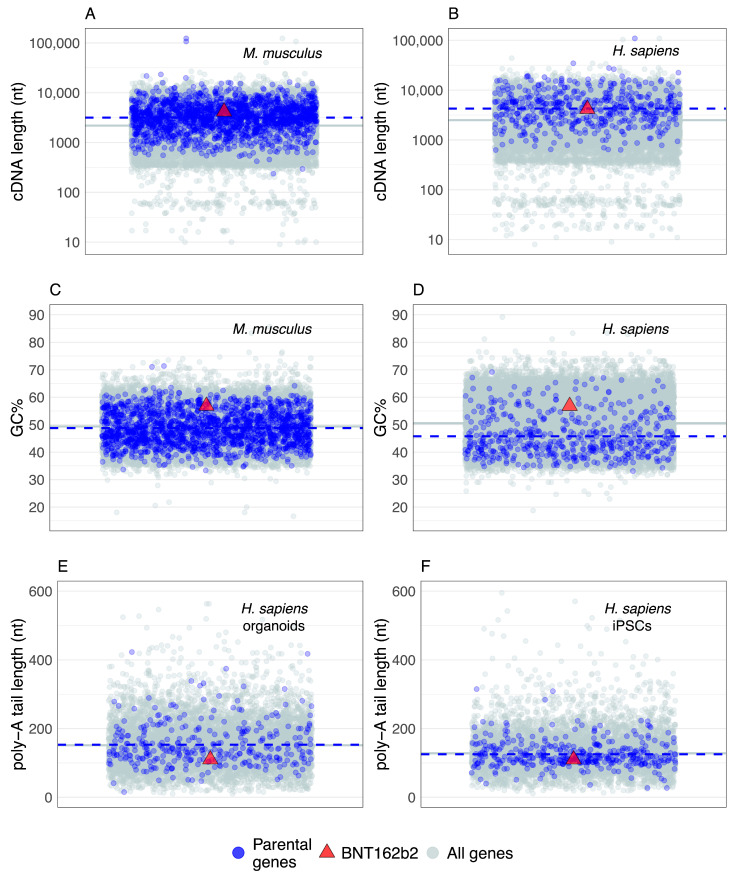
The basic sequence properties of BNT162b2 mRNA are within the range of parental genes that generate retrocopies. The jitter plots show parental genes (blue dots) and all genes (gray dots) randomly distributed along the x-axis. The red triangles show the BNT162b2 mRNA values. The significance of the difference between the parental genes average (blue dashed line) and the all genes average (gray solid line) was tested using a permutation test (two-tailed, 10^6^ permutations). The initial lists contained 503 human [37] and 1663 mouse parental gene names [40]. All mouse and 496 human parental gene names were successfully linked to the sequence data. Poly-A tail lengths were obtained for 7760 (organoids, replicate 1) and 9132 (iPSCs, replicate 1) human genes by averaging multiple estimates per gene [89]. (**A**) The comparison of cDNA lengths in mice (*p* = 0; 22,770 all genes, 1663 parental genes, Ensembl GRCm38.86). (**B**) The comparison of cDNA lengths in humans (*p* = 0; 22,964 all genes, 496 parental genes, Ensemble GRCh38.86). (**C**) The comparison of GC content in mice (*p* = 0.00021; 22,770 all genes, 1663 parental genes, Ensembl GRCm38.86). (**D**) The comparison of GC contents in humans (*p* = 0; 22,964 all genes, 498 parental genes, Ensemble GRCh38.86). (**E**) The comparison of poly-A tail lengths in human-iPSC-derived cerebral organoids (*p* = 0.69; 7760 all genes, 330 parental genes, Ensemble GRCh38.84). (**F**) The comparison of poly-A tail lengths in human induced pluripotent stem cells (iPSCs) (*p* = 0.26; 9132 all genes, 369 parental genes, Ensemble GRCh38.84).

The current estimate of 503 parental genes in humans [37] is lower than in mice, where 1663 of them are recovered [40]. However, a study in mice that used an improved retrocopy detection pipeline and higher sequencing depths found that the number of parental genes has not reached saturation; thus, the actual number of parental genes should be expected to be higher, especially in humans [40]. Regardless of this inherent incompleteness, the available datasets showed that both mouse and human parental genes have a broad distribution of mRNA lengths (Figure 2A,B). It is also evident that the mRNAs of parental genes tend to have slightly longer sequences than the average for all protein-coding genes (Figure 2A,B). Under the caveat that I considered here only the longest splicing variant per gene, and that shorter and intronless genes might be overlooked in the retrocopy/parental gene detection pipelines, this result revealed that L1-mediated retroposition in *trans* is modulated to some extent by parental gene mRNA sequence length. In any case, the sequence length of BNT162b2 mRNA falls very close to the average mRNA length of parental genes (Figure 2A,B), indicating that the sequence length of BNT162b2 mRNA will likely not be an obstacle to retroposition.

To improve their translation and stability, vaccine mRNAs are frequently sequence- and/or codon-optimized [1,6,56,150] and this optimization could affect the GC content. Hence, I explored the GC content in mice and humans to see whether BNT162b2 mRNA is outside the range of parental genes. Similar to the mRNA length analysis, the GC content of parental genes shows a broad range of values (Figure 2C,D). In mice, the average GC content of parental genes is almost equal to the genome average (Figure 2C), whereas, in humans, parental genes tend to have slightly lower values (Figure 2D). Although the GC content of BNT162b2 mRNA is higher than the average of parental genes, it is well within their range (Figure 2C,D); thus it is unlikely that the peculiarities of the BNT162b2 GC content will prevent its retroposition.

The mRNA sequences analyzed so far correspond to bioinformatic cDNA sequences; i.e., coding sequence plus untranslated regions, excluding the poly-A tail. Commonly, poly-A tails are not considered in genome-based analyses because they are post-transcriptionally added and it was technically challenging to precisely recover their nucleotide sequence. However, poly-A tail sequencing approaches at the transcriptome scale are continuously improving and recently produced datasets provide an opportunity to gain insight into the distribution of their lengths [89]. Here, I explored poly-A tail lengths estimated using FLAM-seq in human induced pluripotent stem cells (iPSCs) and iPSCs-derived cerebral organoids [89]. I found no difference between the average poly-A tail lengths of known parental genes and all coding genes (Figure 1E,F). The distribution range of parental gene poly-A tail lengths is rather broad (Figure 1E,F), indicating that L1 machinery is mostly insensitive to the variation in poly-A tail lengths. The BNT162b2 poly-A tail with 110 nucleotides is well within the range of these values; therefore, no specific difficulties in retroposition regarding the poly-A tail length are expected. At this point, it is worth mentioning that the poly-A tail is present in other mRNA vaccine candidates as well [5,151,152].

This simple ad hoc comparative analysis, which covers the length, GC content and poly-A tail length of parental genes that actively produce retrocopies in extant populations (Figure 2), could be expanded by considering other datasets and sequence traits, or by using more sophisticated analytical approaches. However, its main purpose is to show that effectively any poly-A tail containing mRNA in human cells, including vaccine mRNAs, has some chance to be integrated into the genome by L1 machinery. It also reveals that is very incautious to assume without testing that mRNA-based therapeutics are integration-safe, e.g., [6,10,23,24,25].

## 7. Pharmacology Aspects

Synthetic mRNAs have rather complex pharmacology that is dependent on their nucleotide sequence, formulation and administration route [10,56,143]. The likelihood of synthetic mRNA genome integration via L1 elements, besides the nucleotide sequence, depends on its distribution in tissues and organs, and eventually on its concentration and stability in the cell cytosol (Table 1). The quantity of synthetic mRNA in a single dose is the initial factor that determines the pharmacokinetics and pharmacodynamics of mRNA vaccines [10,143]; hence, it is helpful to consider the declared values for BNT162b2 and mRNA-1273. In a single 30 μg BNT162b2 dose [1,140], there are around 1.3 × 10^13^ synthetic mRNA molecules. If we ignore the loss of vaccine mRNAs on the route to the cytosol and assume their homogenous distribution among roughly 3 × 10^12^ nucleated cells in the human body [153], then every nucleated cell could receive about 26 mRNA copies. This is a substantial amount if compared to the expressed human protein-coding genes that have on average 25 mRNA copies per cell [154]. These values show that the quantity of vaccine mRNA delivered in a single dose of BNT162b2 is large enough to theoretically reprogram the transcriptome of every single human cell that in principle can undergo retroposition. The undisclosed sequence of mRNA-1273 vaccine prevents a similar calculation, but under the assumption that its sequence length and nucleotide composition are comparable to BNT162b2 [2,5,56], the number of mRNA molecules per nucleated cell is possibly even higher because a single dose of mRNA-1273 vaccine contains 100 μg of synthetic mRNA [2,56]. This calculation provides the theoretical upper bound of vaccine mRNA cellular uptake; however, the lower bound is much more challenging to estimate due to the complex pharmacology of synthetic mRNAs [10] and the rather limited data in the literature [1,2,56,140].

After intramuscular inoculation, BNT162b2 and mRNA-1273 mRNA molecules should reach the cell cytosol where they are translated to SARS-CoV-2 spike proteins, which eventually elicit the protective immune response [1,2,56,143,155]. On this road from the entry site to the cell cytosol, some naked and unmodified mRNAs would be mostly degraded by the omnipresent extracellular ribonucleases [5,6,10,156]. The remaining mRNAs that eventually enter the cell through endocytosis predominantly end up entrapped in endosomes and degrade over time [10,56,155,156]. On top of this, naked mRNAs with unmodified nucleosides are detected in the endosome and cytosol by pattern recognition receptors, which, by triggering the interferon signaling and other pathways, promote RNA degradation, induce inflammation and inhibit translation and replication [5,10,56]. Therefore, even if some external mRNAs reach the cytosol, their half-life should be largely compromised. These multiple innate immunity mechanisms against external RNAs show that eukaryotic cells are under strong selective pressure to avoid transcriptome reprogramming. By preventing the entry and activity of external mRNAs in the cytosol, these protective mechanisms also largely preclude the possible interaction of external mRNAs and endogenous L1 machinery and consequently lower the chances that some exogenous mRNAs undergo retroposition.

However, for mRNA vaccines to be effective, they must overcome these innate defense mechanisms against exogenous RNAs, reach the cytosol and have to be efficiently translated by ribosomes [6,10]. In the case of BNT162b2 and mRNA-1273 vaccines, this is achieved by elaborate sequence optimizations and nucleoside modifications that stabilize synthetic mRNAs and make them largely invisible to innate defense mechanisms (Table 1) [1,2,6,10,56]. To further protect them from the harsh extracellular environments, they are formulated in lipid nanoparticles (LNPs) that facilitate their cellular uptake and cytosol entry via endosomal escape [1,2,10,56,143]. It is important to note that these remarkable engineering achievements that improve vaccine mRNA cytosol delivery inadvertently increase the chances of vaccine mRNA retroposition (Table 1, Figure 1C). This shortcoming stems from the fact that, in principle, any improvement in the vaccine mRNA cytosol delivery increases the probability of interaction with the endogenous L1 machinery. Nevertheless, regardless of the increased stability and LNP formulation of vaccine mRNAs, a substantial fraction of the initial dose is degraded and will never reach the cytosol [143,155]. Unfortunately, accessible information in the public domain on BNT162b2 and mRNA-1273 does not reveal which percentage of the initial vaccine mRNA dose becomes bioavailable in the cytosol [1,2,143]. In any case, any further improvement in the cytosol delivery of vaccine mRNAs, which is a heavily pursued goal in the mRNA vaccinology field [6,10,143,155,157,158], will concomitantly increase the chances of L1-mediated retroposition (Figure 1C, Table 1).

Every mRNA molecule in the cytosol will eventually decay through one of many degradation pathways [159,160]. In contrast to exogenous vaccine mRNAs that once degraded are not replaced [6,10,157], the levels of endogenous mRNAs are controlled by the interplay between transcription and decay [159,160]. If all other parameters are ignored, this would mean that the probability of L1-mediated retroposition is higher for an endogenous gene with typical levels of expression than for a vaccine mRNA that is transiently present in the cell. However, several additional factors increase the chances of vaccine mRNA retroposition. The number of received doses per individual directly increases the chance of retroposition because it prolongs the time for the encounter of vaccine mRNA with L1 machinery (Table 1). Initially, BNT162b2 and mRNA-1273 were administered intramuscularly as a series of two doses, three weeks and one month apart, respectively [1,2,56]. However, later on, additional booster vaccinations were introduced [141,142]. It is evident that any increase in the number of required doses further increases the chances of vaccine mRNA retroposition. This could be a particularly prominent problem if the mRNA vaccines would require long-term recurrent applications, as in the case of the current seasonal vaccination program against influenza [142,161].

An additional property that influences the likelihood of vaccine mRNA genome integration is the stability of vaccine mRNA molecules. The turnover of endogenous mRNA molecules in eukaryotic cells shows great variability, with an estimated average half-life of around 7 h [138]. The precise measurements of the vaccine mRNA half-life in cells are not publicly available [1,2], but it is clear that the sequence and codon optimization of vaccine mRNAs increases their functional half-life with an aim to improve their translation efficiency [6,10,27,56,138,139]. Undoubtedly, this prolonged functional half-life increases the chances that vaccine mRNAs encounter L1 machinery and eventually retropose into the genome (Table 1). In addition, it remains unexplored how vaccine mRNAs interact with ribonucleoprotein granules that participate in the regulation of mRNA storage and decay [28,159,162,163], as well as with the cytoplasm-residing L1 ribonucleoprotein particles [164].

## 8. Biodistribution Profiles

A biodistribution profile is another important parameter that determines the likelihood of vaccine mRNA genome integration because the activity of L1 elements differs between the cells, tissues and organs [99,100,114]. Interestingly, direct biodistribution studies have not been conducted for the BNT162b2 vaccine [1]. However, surrogate studies in mice and rats indicate distribution, in different quantities, from the injection site to most tissues, including liver, adrenal glands, spleen and gonads [1]. Direct distribution and pharmacokinetic studies for the mRNA-1273 vaccine were also not conducted, but studies in rats using the same LNPs and a cocktail of mRNAs encoding cytomegalovirus antigens indicate that these mRNAs, with the exception of kidney, could be detected at varying levels in all examined tissues, including the injection site muscle, proximal and distal lymph nodes, spleen, eyes, heart, lung, brain and testis [2]. Notably, the distribution of mRNA to ovaries was not tested because no female rats were included in this study, as explained in the regulatory documents [2]. Obviously, these surrogate biodistribution profiles substantially overlap with organs known to show the activity of L1 elements, such as liver [127], spleen [106], brain [60,99,117,118,119,120,121], adrenal glands [106], muscles [104,131,165] and gonads [96,105,106,109,112] (Table 1).

If the quantity of vaccine mRNA in a single dose of BNT162b2 or mRNA-1273 is considered, these neither strictly localized nor fully systemic distribution patterns suggest that in some tissues, vaccine mRNA likely accumulates in rather high concentrations, with the potential to saturate the exogenous mRNA uptake capacity of recipient cells [10,166]. To more precisely evaluate the probability of L1 mediated retroposition, it is important to understand which cell types can uptake vaccine mRNA. Dendritic cells and macrophages present at the inoculation site and draining nodes are, according to the regulatory body, the two principal cell types targeted by BNT162b2 and mRNA-1273 vaccines [167]. However, the assessment report for the BNT162b2 vaccine stated that it is unknown whether cells other than professional antigen-presenting cells (APCs) may transiently express the vaccine-derived spike protein [1]. Similarly, the mRNA-1273 vaccine assessment report declared that the delivered vaccine mRNA is mainly expressed by macrophages and dendritic cells [2]. This apparently reveals that the mRNA-1273 is expressed in some other cell types as well. It is also indicative that the mechanisms of action that would drive BNT162b2 and mRNA-1273 exclusively/preferentially to dendritic cells and macrophages, if they exist, were not explained in these documents [1,2,167].

Although macrophages and dendritic cells, as professional antigen-presenting cells (APCs), are specialized in sampling their environment, essentially all nucleated cells are endocytosis competent. The evidence from several studies indicates that the cellular uptake of the mRNA LNPs relies on the apolipoprotein E (ApoE) binding to LNPs and their subsequent endocytosis that is facilitated by low-density lipoprotein (LDL) receptors [56,166,168,169]. Since ApoE, LDL and LDL-like receptors are expressed by many cell types throughout the body [170,171], it could be expected that APCs are not the only cell types that internalize mRNA LNPs [56,169]. For example, some studies indicate that myocytes, epithelial cells and fibroblasts take up vaccine mRNA and contribute to its expression [56,172,173,174]. These considerations suggest that cell types other than dendritic cells and macrophages most likely internalize BNT162b2 and mRNA-1273 vaccine mRNAs and that the potential encounter of L1 machinery and vaccine mRNAs may occur in diverse cell types within the broad range of tissues (Table 1).

Another level of complexity in the transport and uptake of LNP-formulated exogenous mRNA arises with the recent finding that, after endocytosis, LNPs containing mRNA are repackaged in late endosomes and secreted back into extracellular space as extracellular vesicles (EVs) (Figure 1C) [57]. These vaccine mRNA EVs (endo-EVs) protect exogenous mRNA in extracellular fluids during in vivo transport to other organs and deliver intact exogenous mRNA to the cytoplasm of the distant recipient cells [57,58,175,176,177]. Because of their small size, vaccine mRNA EVs are less visible than LNPs to innate immunity mechanisms and can pass through the vascular endothelium and the extracellular matrix [57,178]. Given that many cell types, including dendritic cells [179] and macrophages [180], secrete EVs, the range of cells and tissues that exogenous mRNAs could reach is substantially broadened compared to the LNPs route only (Figure 1C). A recent study showed that L1 mRNAs in cultured cells could also be packaged into EVs, delivered via EVs to recipient cells and retroposed into their genome (Figure 1A) [53]. Together, this suggests that the dynamics of EVs substantially raise the odds for the interaction between active L1 elements and vaccine mRNAs (Figure 1C, Table 1).

The possibility of vaccine mRNA genome integration in somatic and germline cells (Figure 1) is not the only adverse effect that should be considered. Theoretically, the vaccine mRNA could also be epigenetically inherited via the sperm RNA cargo [181,182,183,184]. This could happen if the testis cells of the male germinative lineage take up LNPs or EVs containing vaccine mRNAs, and if these mRNAs then end up in spermatozoa [182,183,185]. Alternatively, during their functional maturation in the epididymis, spermatozoa could potentially actively internalize vaccine mRNAs delivered by epididymal EVs [184,185]. The presence and integration of vaccine mRNAs in human spermatozoa could be relatively easily tested because the semen of vaccinated men, compared to other integration-relevant tissues, is a relatively accessible body fluid.

## 9. Final Remarks

There are some further points that should be mentioned. Several papers reported that the infection of human cells by viruses, including SARS-CoV-2, increases the activity of their endogenous L1 retroelements [54,55,144,145,146], which is consistent with the presumed environmental modulation of L1 activity [114]. These findings suggest that, paradoxically, mRNA vaccination during active or after resolved viral infection might increase the chances of vaccine mRNA genome integration. The COVID-19 vaccine mRNAs code for the SARS-CoV-2 spike protein [56]; therefore, it is important to know whether there is any evidence that SARS-CoV-2 mRNAs could integrate into the genome. Indeed, a recent study shows that upon infection, SARS-CoV-2 subgenomic mRNAs can be reverse-transcribed by L1 elements and integrated into the genome of infected cells [54], although another study partly challenges these results [55]. Interestingly, fragments of mRNAs closer to the 3′ end of the SARS-CoV-2 genome, including the spike mRNA, are more frequently integrated into the cell DNA than the sequences closer to the 5′ end [54]. This integration bias could be related to the differences in the abundance of SARS-CoV-2 subgenomic mRNAs [186], as suggested by the authors [54]. However, it could also reflect the nested architecture of subgenomic mRNAs [186] coupled with the mechanism of L1 retroposition that relies on the poly-A tail [51] and is prone to truncate transcripts with increasing distance from the 3′ end.

L1 retrotransposon activity is closely linked with replication [47,86,187,188], and it was suggested that the retroposition of cellular mRNAs is coupled to cell divisions [37,64]. This implies that the risk of vaccine mRNA genome integration might be increased in human proliferating cell populations. The biodistribution profiles of vaccine mRNA are not available for tumors; however, increased replication activity, coupled with elevated L1 retrotransposition in tumor cells [84], make them a favorable environment for possible vaccine mRNA genome integration (Table 1). In this regard, it would be very informative to test the biodistribution profile of mRNA vaccines in murine tumor models and to look for eventual somatic retroposition events. Indeed, around six months after the preprint version of this paper was made public, an experimental study was published that reported the high-level uptake of BNT162b2 and its reverse transcription in a human liver tumor cell line [189].

At first glance, it appears that the application of mRNA vaccines could not alter the primary retroposition rates at the individual and population levels. The underlying reason is that vaccine mRNAs are not directly mutagenic and that their route to potential genome integration hinges on the endogenous cellular mechanisms, i.e., the activity of L1 elements that continuously operate on the available mRNA pool. Nevertheless, the possible change in primary retroposition rates should not be immediately dismissed because it cannot be excluded without testing that vaccination with LNP-formulated mRNAs does not modulate L1 activity. As already explained, it is well established that many exogenous factors modify L1 activity [114], including viral infections [54,55,144,145,146]; therefore, the impact of mRNA vaccination should also be evaluated in this regard.

On the other hand, it is apparent that eventual vaccine mRNA genome integration broadens the spectrum of conceivable sequences that could be retrocopied (Figure 1). Our cells evolved under mutational pressure that came from the activity of L1 elements that generate retrocopies of our native genes [37,40]. However, the transfection of human cells with exogenous and artificially modified mRNAs, which have the potential to be retrocopied into the genome (Figure 1C), extends the standard mutational sequence space to the realm of transgenic modifications. It is rather clear that any possibility of transgenesis in humans has ethical concerns that should be properly addressed. This raises two questions: Who is responsible for testing the likelihood of vaccine mRNA retroposition, and who will be responsible for eventual genome modifications resulting from the application of emergency-use mRNA vaccines? The answers to these questions are, without doubt, of outstanding importance for society at large.

The retroposition of a vaccine mRNA molecule is, in principle, a random event that can occur in any transfected cell that shows the activity of L1 elements (Figure 1C). The clonal expansion of a new retrocopy largely depends on its phenotypic effects and the pre-existing proliferative capacity of the mutated cell. On one extreme, a vaccine mRNA retrocopy that directly inactivates an essential gene [113,190] would result in cell death that would preclude any further spread of that retrocopy. However, a retrocopy that is moderately deleterious or neutral [76,191] and has emerged in a cell with high proliferative potential has good odds to be propagated to a large number of descendant cells. In adults, the proliferative capacity of many cells in the soma is considerably limited [191,192] and it further drops with aging [193]. This implies that the vaccine mRNA retrocopy mosaicism in the adult soma should be largely restricted to smaller cell clusters or individual cells. Nevertheless, a retroposition event in a progenitor cell, an adult stem cell [194] or a premalignant cell [193] would lead to clonal expansion of the retrocopy in much larger chunks of somatic tissue.

In contrast to the relatively confined effects of somatic retroposition, a possible heritable vaccine mRNA retroposition event would have a more far-reaching impact by rendering fully transgenic individuals. The hypothetical vaccine mRNA retrocopy with heritable potential could occur in germinative cells or in the pluripotent cells of early embryos [113]. As already discussed above, the documents of regulatory agencies state that the surrogate biodistribution studies reported the distribution of LNP-formulated mRNA to gonads [1,2], which are known to display the activity of L1 elements [96,99,105,106,109,110,111,112]. On the other hand, vaccine mRNA stored in the sperm RNA cargo could hypothetically reach the pluripotent cells of early embryos, which are the hotspots of L1 activity [93,94,95,97,107,108] and undergo retroposition there. This, in turn, could result in somatic mosaicism, where a substantial part of cells in an individual could become transgenic, and if the gonads are also affected, the retrocopy could become heritable [97,113].

The phenotype of a vaccine mRNA retrocopy will depend, among other factors, on the number and identity of cells that become transgenic, the insertion locus, completeness of the inserted sequence, direction of the insertion, peculiarities of the recipient genome and the expression potential of the retrocopy. Although native mRNAs lack transcription-driving elements, it is well established that most of their retrocopies show evidence of transcription [38,40,41]; hence, it could be expected that a hypothetical vaccine mRNA retrocopy would also have good chances to be expressed. Many expressed retrocopies of native genes tend to have a strong negative impact on fitness and are, therefore, relatively quickly purged from the population [40]. It was suggested that these deleterious effects of expressed retrocopies are often related to the interference with their parental genes [40]. Since a hypothetical vaccine mRNA retrocopy does not have a parental gene in the host genome (Figure 1C), effects related to the expression interference between the retrocopy and its parental gene are not possible. However, an expressed retrocopy of vaccine mRNA could interact in unpredictable ways with the host immune system, later viral infections, vaccine mRNAs received in subsequent administration rounds and native mRNAs. For instance, the antisense-transcribed UTR regions of a vaccine mRNA retrocopy could potentially silence complementary transcripts of human globin genes [43,195,196,197].

## 10. Conclusions

Current engineering strategies [150] and declared future directions [150,198] for the improvement of mRNA vaccines do not consider the possibility of vaccine mRNA genome integration via L1 retroelements native to human cells. This is at odds with the knowledge base on the biology of L1-mediated retroposition and its role in the genetics, development and evolution of humans. Why this risk is overlooked is even more obscure given that mRNA retroposition is a biomedically recognized phenomenon outside vaccinology [44,49,62,65,66,68,69,77,79,80,83]. To alleviate these discrepancies between the fields, it would be critical to design and perform experimental studies on animal models that aim to detect the existence of vaccine mRNA retrocopies and estimate their retroposition frequencies. In this endeavor, the single-cell sequencing of various tissues will likely play a pivotal role [191]. As the retroposition propensity via L1 retroelements is sequence-dependent, it would be advisable to independently test every mRNA therapeutic candidate. This information could then guide further vaccine mRNA refinements in the direction of avoiding active L1 cellular environments [199] or by improving their resilience to the L1 machinery capture [102].

Every technology is a double-edged sword and mRNA therapeutics are not an exception. In this complex COVID-19 crisis, it is essential that all pros and cons of control measures, procedures, treatments, prophylaxis and vaccine technologies are continually openly discussed and cautiously evaluated from many angles [200]. An encouraging example in this direction is the recently published papers that, in a balanced way, discuss the largely ignored negative aspects of COVID-19 pandemic control measures and practices on the overall human microbiome [201], neonatal microbiome [202] and immunity [203]. I hope that the possible interplay between mRNA vaccines and L1 elements presented here will also provoke debate and attract the attention of researchers in a broad range of disciplines, e.g., [204].

Whether the current vaccine mRNAs could integrate into the genome, and by which frequency, has to be ultimately demonstrated using experiments. However, it remains puzzling why and how the mRNA vaccinology field neglected the retroposition biology of L1 retroelements and its theoretical links to possible vaccine mRNA retroposition, especially when one considers the volume, visibility and significance of the L1 [44,45,60,83,84,85,117,131] and retroposition research [36,37,38,39,40,41,45,46,49,60,66,68,77,80]. The mRNA vaccinology field started its development more than 30 years ago [11,31] and L1 retroelements in humans have been studied for more than 40 years [205,206] but obviously without any crosstalk between the two fields. This awkward silo effect points to the fact that, on some occasions, the structural drawbacks of contemporary science, despite its amassment, globalization and unprecedented dissemination, are deeper than we are willing to admit. Unfortunately, all of this creates an impression that L1-driven retroposition is a kind of taboo topic in mRNA vaccinology.

I conclude that the broadly reiterated statement that mRNA-based therapeutics could not impact genomes is an unfounded assumption of unclear origin. This implies that the current mRNA vaccine evaluations, which lack studies that specifically address genome integration, are insufficient to declare their genome integration safety. We should not forget that mRNAs are information-bearing molecules that can, in principle, encode any information [207]. From this perspective, an mRNA vaccination could be viewed as a sort of “molecular tattooing”. It is, therefore, important that the exact nucleotide sequences of mRNA vaccines are disclosed and easily publicly accessible, including product information documents [208,209], to allow for unambiguous and independent tracking of possible vaccine mRNA integration in the somatic and germinative genomes of already vaccinated people and their progeny.

## Figures and Tables

**Table 1:** The list of factors that increase the possibility of vaccine mRNA retroposition by L1 elements.

**mRNA Vaccine Features**	**References ^1^**
Native mRNA architecture	[6,10,28,133]
3′ poly-A tail	[51,52,132]
m1Ψ modification	[20,134,135,136]
Improved stability, half-life and translational efficiency	[6,10,27,56,133,138,139]
mRNA concentration per dose	[1,140]
Recurrent application	[1,2,56,141,142]
Lipid nanoparticle formulation	[1,2,10,56,143]
Cytosol delivery	[5,6,10,56]
Biodistribution	[1,2]
Extracellular vesicles repackaging	[53,57]
**Other Factors**	**References** ^1^
Increased cell proliferation rates	[37,64,84]
Aging	[104,131]
Viral infection	[54,55,144,145,146]

^1^ Some of the relevant references that describe these factors. For a full discussion, see the main text.

## Data Availability

Publicly available datasets were analyzed in this study. The mouse and human sequence data is available in the Ensembl repository: (http://ftp.ensembl.org/pub/, accessed on 30 December 2020). The lists of parental genes are available in the supplementary material of the respective references [37,40]. The human poly-A tail data is available in the NCBI GEO database: (https://www.ncbi.nlm.nih.gov/geo/query/acc.cgi?acc=GSE126465, accessed on 30 December 2020).
